# Longitudinal effects of dog ownership, dog acquisition, and dog loss on children’s movement behaviours: findings from the PLAYCE cohort study

**DOI:** 10.1186/s12966-023-01544-9

**Published:** 2024-01-30

**Authors:** Emma K. Adams, Kevin Murray, Stewart G. Trost, Hayley Christian

**Affiliations:** 1 Telethon Kids Institute, University of Western Australia, 35 Stirling Hwy, Perth, Western Australia 6009 Australia; 2 School of Population and Global Health, University of Western Australia, 35 Stirling Hwy, Perth, Western Australia 6009 Australia; 3 School of Human Movement and Nutrition Sciences, University of Queensland, Brisbane, Queensland 4072 Australia

**Keywords:** Dog ownership, Physical activity, Children, Longitudinal, Cohort, Dog acquisition, Dog loss, Preschool

## Abstract

**Introduction:**

Regular physical activity is important for children’s physical and mental health, yet many children do not achieve recommended amounts of physical activity. Dog ownership has been associated with increased physical activity in children, however, there have been no longitudinal studies examining this relationship. This study used data from the Play Spaces and Environments for Children’s Physical Activity (PLAYCE) cohort study to examine the longitudinal effects of dog ownership status on children’s movement behaviours.

**Methods:**

Change in dog ownership from preschool (wave 1, age 2–5) to fulltime school (wave 2, age 5–7) was used as a natural experiment with four distinct dog ownership groups: continuing non-dog owners (*n* = 307), continuing dog owners (*n* = 204), dog acquired (*n* = 58), and dog loss (*n* = 31; total *n* = 600). Daily movement behaviours, including physical activity, sedentary time, sleep, and screen time, were measured using accelerometry and parent-report surveys. Differences between groups over time and by sex were tested using linear mixed effects regression models.

**Results:**

Girls who acquired a dog increased their light intensity activities and games by 52.0 min/day (95%CI 7.9, 96.0) and girls who lost a dog decreased their light intensity activities and games by 62.1 min/day (95%CI -119.3, -4.9) compared to no change among non-dog owners. Girls and boys who acquired a dog increased their unstructured physical activity by 6.8 (95%CI 3.2, 10.3) and 7.1 (95%CI 3.9, 10.3) occasions/week, compared to no changes among non-dog owners. Girls and boys who lost a dog reduced their unstructured physical activity by 10.2 (95%CI -15.0, -5.3) and 7.7 (95%CI -12.0, -3.5) occasions/week. Girls who lost a dog decreased their total physical activity by 46.3 min/day (95%CI -107.5, 14.8) compared to no change among non-dog owners. Continuing dog ownership was typically not associated with movement behaviours. Dog ownership group was not associated with changes in sleep and had mixed associations with screen time.

**Conclusion:**

The positive influence of dog ownership on children’s physical activity begins in early childhood and differs by child sex. Further research should examine the specific contributions dog-facilitated physical activity makes to children’s overall physical activity, including the intensity and duration of dog walking and play.

**Supplementary Information:**

The online version contains supplementary material available at 10.1186/s12966-023-01544-9.

## Introduction

Regular physical activity among children supports healthy weight status, improves musculoskeletal health, cardio-respiratory fitness, and mental wellbeing, and reduces chronic disease risk [1–5]. International guidelines recommend children aged 1 to 4 accumulate 180 min of total physical activity each day, including 60 min of moderate-to-vigorous intensity physical activity (energetic play) for children aged 3 to 4 [6]. As well, children aged 5 to 17 should accumulate 60 min of moderate-to-vigorous intensity physical activity each day [5]. In recognition of the importance of the whole days’ movement behaviours on child health and development [7–9] the guidelines also provide age-specific recommendations for sedentary time, screen time, and sleep [5, 6, 10]. Australia [11, 12], and other countries [10, 13, 14], have similar age-specific movement guidelines. However, a large proportion of children do not meet physical activity or screen time recommendations for their age group [15, 16].

Around 40–50% of Australian households have a dog [17–19] and there is growing evidence dog ownership is associated with higher levels of physical activity in children [20–26]. Moreover, within dog-owning children and adolescents, greater frequencies of dog play and dog walking are associated with increased physical activity [21, 25, 27] and increased likelihood of meeting physical activity recommendations [25]. Of the few studies examining the relationship between dog ownership and children’s screen time [20, 21, 26, 28] or sleep [21, 26], no associations with screen time have been reported and only one study found a positive association with sleep (during the COVID-19 pandemic) [26].

However, while the effects of dog walking on children’s physical activity have been investigated in two pilot trials [29, 30], no longitudinal studies have been conducted to examine the causal relationship between dog ownership and children’s movement behaviours. Given the responsibilities of dog ownership, particularly a dog’s daily exercise needs (i.e., dog walking), it is possible that families who are already more physically active acquire dogs since dog walking may more easily fit into their lifestyles [31]. Conversely, people who acquire dogs may increase their physical activity via dog walking because owning a dog provides a sense of motivation and obligation to be active with their dog [32]. Even among adults there are few longitudinal studies on the effects of dog acquisition on physical activity [31, 33–36]. Five studies report increases in walking practices [31, 33, 34], daily steps [35, 36], or moderate-to-vigorous physical activity [36] following dog acquisition. However, most longitudinal studies have been limited by small sample sizes and lack of adjustment for potential confounding variables [33–36], and two had no comparison group [34, 36]. Furthermore, if dog acquisition increases physical activity, it is also plausible losing a dog decreases physical activity, but this has not been explored in research. To date, it is unknown if dog acquisition and/or dog loss affect changes in young children’s movement behaviours.

A 2013 systematic review of studies investigating the effects of dog ownership on physical activity found only four of 29 studies used device-measured physical activity [37]. Two of these studies included children [22, 23], and since that review, just one additional study among children has incorporated device-measured physical activity [21]. Importantly, all three of these studies used traditional cut-point data processing methods to derive minutes of time spent in varying intensities of physical activity. Cut-point data processing methods have substantial limitations resulting in misclassification of intensity for large proportions of data [38–41]. To address these limitations, researchers have begun implementing machine learning models to predict physical activity type and intensity [42, 43].

Research on the longitudinal effects of dog ownership status presents ethical challenges [44, 45]. For example, it is unethical to implement a randomised controlled trial where families are randomly assigned to a dog ownership condition. Thus, to compare movement behaviours between varying dog ownership conditions requires either using existing datasets where dog ownership functions as a natural experiment or recruiting future dog owners during the dog adoption or purchase process and recruiting a similar sample of non-dog owners as a comparison group. Studies using the latter design are expensive to conduct and have had small sample sizes and consequently may be underpowered and subject to selection bias. Therefore, the aim of this study was to use longitudinal data from the Play Spaces and Environments for Children’s Physical Activity (PLAYCE) cohort study to examine the effects of dog ownership, dog acquisition, and dog loss compared with non-dog ownership on young children’s movement behaviours.

## Methods

### Study design and sample

**Fig. 1:**
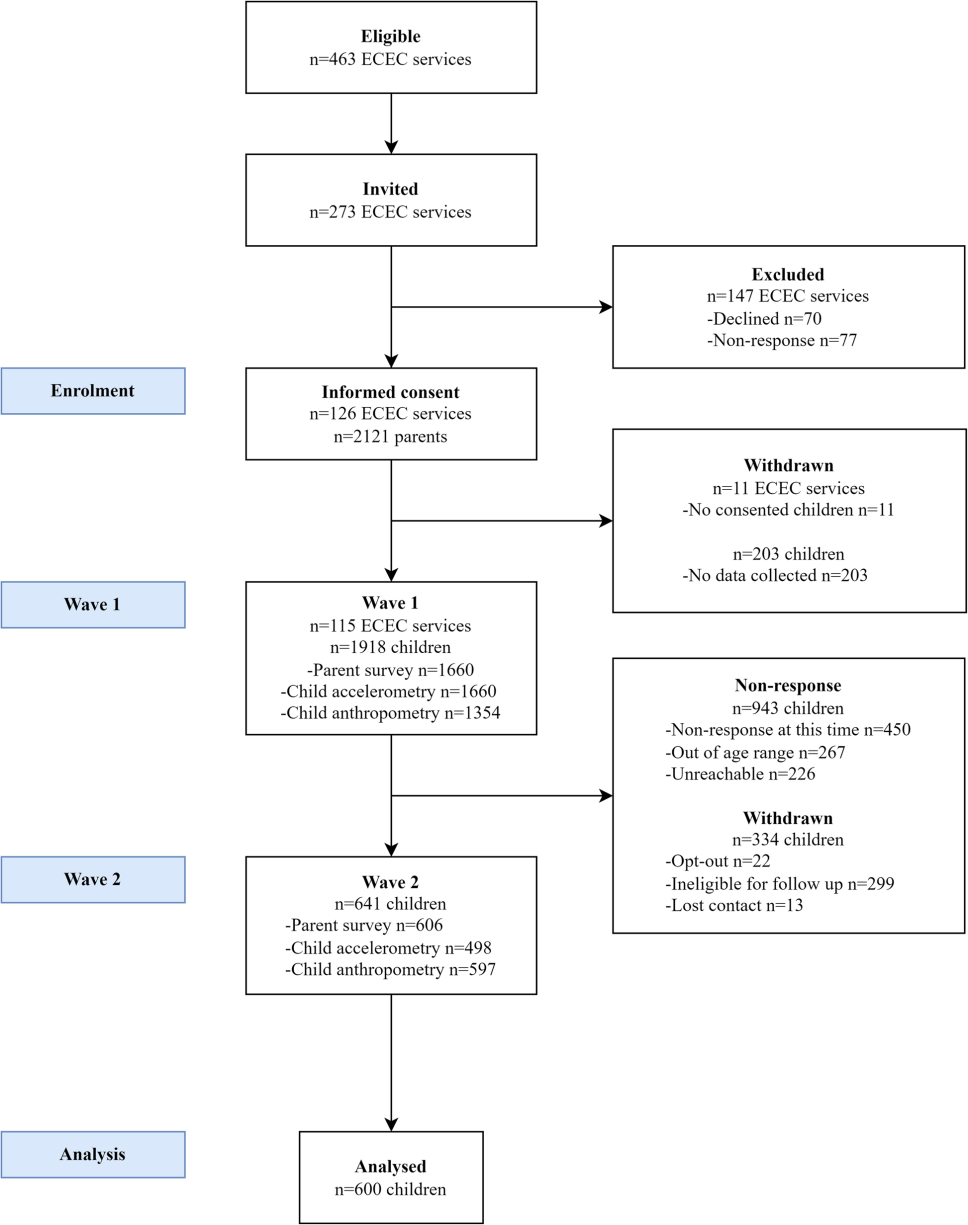
PLAYCE cohort flow diagram

The PLAYCE cohort study commenced as an observational study in Perth, Western Australia. Children were recruited through early childhood education and care (ECEC) services, which were selected based on size and socio-economic status [46]. Parents from selected services were invited to provide written informed consent for themselves and their child to participate. These baseline (wave 1) data were collected from April 2015 to April 2018 for 1,918 children aged 2 to 5 years old (Fig. 1). All children were invited to participate in follow-up (wave 2) data collection as they transitioned to full time school (Pre-Primary and Year 1). Children were ineligible at wave 2 if they had not yet transitioned to full time school, were more than 8.5 years old, or were no longer living in the study region. Wave 2 data were from October 2018 to June 2021 for 641 children aged 5 to 7 years. Children were included in the current study if they had dog ownership data at both wave 1 and wave 2 (*n* = 600). Dog ownership group was treated as a natural experiment with four mutually exclusive groups: no dog ownership at wave 1 and wave 2 (non-dog owners; control group), dog ownership at wave 1 and wave 2 (dog owners), change to dog ownership from wave 1 to wave 2 (dog acquired), and change to non-dog ownership from wave 1 to wave 2 (dog loss). Of the sample of 600 children, 307 (51.2%) were in the non-dog owner group, 204 (34.0%) in the dog owner group, 58 (9.7%) in the dog acquired group, and 31 (5.2%) in the dog loss group.

The protocol for the original PLAYCE observational study [46] has been published previously. Ethics approval was provided by the University of Western Australia Human Research Ethics Committee (RA/4/1/7417 and 2020/ET000353). A STROBE checklist for the present study is provided in Additional File 1.

### Measures

#### Device-measured physical activity

Children’s physical activity was measured using ActiGraph GT3X + accelerometers (ActiGraph Corporation, Pensacola, FL USA) worn on the hip during waking hours for seven consecutive days. Raw accelerometer data (30 Hz) was processed using a random forest machine learning model for preschool-aged children developed by Ahmadi and colleagues [43] to estimate daily time spent being sedentary (sitting and lying down), light intensity activities and games (slow walking or “pottering about”, standing, standing arts and crafts), walking, running, and moderate-to-vigorous activities and games (active games with balls, riding scooters/tricycles). Non-wear periods were identified by summing the time periods in which the standard deviation of the accelerometer signal vector magnitude was < 13 mg for >  = 30 consecutive minutes [47]. In a free-living evaluation, the random forest model exhibited significantly higher agreement with measured physical activity intensity than cut-point methods and exhibited evidence of equivalence with directly observed time in sedentary activity, light-intensity physical activity, and moderate-to-vigorous physical activity [42]. Daily ‘energetic play’ (moderate-to-vigorous intensity physical activity) was calculated as the sum of walking, running, and moderate-to-vigorous activities and games. Daily total minutes of physical activity was calculated as the sum of light intensity activities and games and energetic play. Children’s accelerometer data were included in the analyses if they had at least three weekdays and one weekend day in which wear time was 8 h or longer (wave 1 *n* = 460, wave 2 *n* = 420; wave 1 and/or wave 2 *n* = 562).

#### Parent-report movement behaviours

Parents completed surveys at wave 1 and wave 2 including measures of family dog ownership (yes/no), screen time (minutes per day), and sleep duration (hours per day). Parents also reported their child’s frequencies of structured and unstructured physical activity using items adapted from the Healthy Active Preschool Years Study [48]. Structured physical activity included five items measuring the number of times per week the child participated in activities such as swimming, dance, and soccer. Total weekly frequency of structured physical activity was calculated by summing responses to each of the items. Unstructured physical activity was measured via 11 items (six-point scale from ‘never/rarely’ to ‘daily’) and included activities such as walking and riding for transport or fun, playing in the yard, and walking and playing with the dog. Total weekly frequency of unstructured physical activity was summed using the mid-point of the responses. Since unstructured physical activity included two items for dog walking and playing with the dog that were not relevant to non-dog owners, a second unstructured physical activity measure was also derived by excluding these two items to produce a measure comparable across the full sample and for examining the contribution of the dog-facilitated activities to total unstructured physical activity.

#### Covariates

Parents reported their level of education, their work status, yard size, dwelling type, and the study child’s age and sex.

#### Sample size and power

This was a secondary analysis of existing data and as such it was not originally designed to look at the research questions of interest. Preliminary calculations for device-based measures suggested we would have sufficient power (> 0.8) to detect 10-min/day differences in energetic play between the dog owner and non-dog owner groups for males and females. However, comparisons involving the dog acquired and dog loss groups had less power. Preliminary calculations for the parent-report measure of unstructured physical activity suggested we would have sufficient power (> 0.8) to detect differences of 7 occasions/week (or an average of one additional occasion/day) for all groups.

#### Analysis

Descriptive statistics for each dog ownership group were computed. Chi-square tests and one-way ANOVAs or the non-parametric equivalent were used to examine differences at wave 1 in child socio-demographics by dog ownership group. PLAYCE study children with only wave 1 data and those with wave 1 and 2 data were compared on their wave 1 characteristics; results are presented in Additional File 2.

Differences in child movement behaviours over time and by dog ownership group were tested using linear mixed effects models (LMMs). Since children’s physical activity is known to vary by sex [49–51], models included interaction terms with child sex. Thus, the LMMs included fixed effects for dog ownership group (non-dog owner = control), time (wave 2 vs. wave 1), child sex (girl vs. boy), and the group-by-time-by-sex interaction and all lower order interactions. LMMs also included random intercept effects to account for repeated measures on individuals. Models were adjusted for wave 1 family covariates (maternal education, maternal work status, dwelling type, yard size) which were selected a priori based on knowledge of potential confounders of dog ownership and child physical activity. To account for individual varying time lengths between wave 1 and wave 2 data collection, the length of follow-up for each child was included as a fixed covariate. To account for wave 2 data collection occurring pre- and during COVID-19, a fixed covariate was included where any child that had data collected on or after March 15, 2020 (the date Western Australia entered a state of emergency) were coded as during COVID-19 and children whose data were collected prior to March 15, 2020 (including all wave 1 data) were coded as pre-COVID-19. Models for accelerometer-derived measures also adjusted for device wear time. Unadjusted model coefficients are presented in Additional File 3. Data were analysed in Stata version 17 using the *mixed* command and included children with data for the dependent variable measured on at least one of the two measurement occasions and data for all confounders. Final analysis samples ranged from 537 to 570 across the range of dependent variables. To aid interpretation of the interaction terms, means at wave 1 and wave 2 were estimated using the *margins* command; *margins* calculates the marginal means of the dependent variables at the specified levels of the independent variables of interest and at the prespecified values of the other covariates in the model.

## Results

### Sample characteristics

Approximately half the sample were girls (48.8%) and the median age at wave 1 was 3.2 years (Table 1). Mother’s education, mother’s work status, dwelling type, and yard size differed significantly between groups. Three-quarters (75.6%) of non-dog owner mothers had a tertiary degree compared to around 60% of mothers from other dog ownership groups. One-third of mothers in the dog acquired (31.0%) and dog loss (32.3%) groups were not in paid employment compared to one-in-seven in the non-dog owner (17.6%) and dog owner (14.8%) groups. Higher proportions of children in the dog owner, dog acquired, and dog loss groups had yards big enough to run around in and lived in a standalone house compared to children in the non-dog owner group.

**Table 1:** Sample characteristics by dog ownership group

Sample characteristics	Total	Non-dog owner	Dog owner	Dog acquired	Dog loss	
	*n* = 600	*n* = 307	*n* = 204	*n* = 58	*n* = 31	
		Median (IQR)	Median (IQR)	Median (IQR)	Median (IQR)	*p*-value
Wave 1 age (years)	3.2 (1.1)	3.2 (1.2)	3.3 (1.0)	3.3 (1.1)	3.1 (0.9)	0.780
Length of follow-up (years)	3.0 (1.0)	2.9 (1.1)	2.9 (1.0)	3.2 (1.2)	3.1 (0.9)	0.112
	*n* (%)	*n* (%)	*n* (%)	*n* (%)	*n* (%)	*p*-value
Child sex	*n* = 600	*n* = 307	*n* = 204	*n* = 58	*n* = 31	
Female	293 (48.8)	150 (48.9)	102 (50.0)	27 (46.6)	14 (45.2)	0.940
Mother’s education	n = 598	n = 307	*n* = 203	*n* = 58	*n* = 31	
Secondary school, trade, or diploma	196 (32.8)	75 (24.4)	85 (42.1)	22 (37.9)	14 (45.2)	< 0.001
Tertiary degree	402 (67.2)	232 (75.6)	117 (57.9)	36 (62.1)	17 (54.8)	
Mother’s work status	*n* = 599	*n* = 307	*n* = 203	*n* = 58	*n* = 31	
Not in paid employment	112 (18.7)	54 (17.6)	30 (14.8)	18 (31.0)	10 (32.3)	0.011
Working full-time	173 (28.9)	81 (26.4)	71 (35.0)	12 (20.7)	9 (29.0)	
Working part-time	314 (52.4)	172 (56.0)	102 (50.3)	28 (48.3)	12 (38.7)	
Yard size	*n* = 574	*n* = 294	*n* = 193	*n* = 56	*n* = 31	
Big enough for running and playing	501 (87.3)	242 (82.3)	180 (93.3)	50 (89.3)	29 (93.6)	0.003
Dwelling type	*n* = 582	*n* = 298	*n* = 196	*n* = 58	*n* = 30	
House	516 (88.7)	251 (84.2)	184 (93.9)	51 (87.9)	30 (100.0)	0.002
Duplex/townhouse/flat/other	66 (11.3)	47 (15.8)	12 (6.1)	7 (12.1)	0 (0.0)	
Wave 1 valid accelerometer data	*n* = 600	*n* = 307	*n* = 204	*n* = 58	*n* = 31	
Yes	460 (76.7)	232 (75.6)	167 (81.9)	41 (70.7)	20 (64.5)	0.072
Wave 1 season^a^	*n* = 460	*n* = 232	*n* = 167	*n* = 41	*n* = 20	
Autumn	134 (29.1)	59 (25.4)	57 (34.1)	11 (26.8)	7 (35.0)	0.664
Winter	122 (26.5)	68 (29.3)	38 (22.8)	12 (29.3)	4 (20.0)	
Spring	159 (34.6)	78 (33.6)	59 (35.3)	15 (36.6)	7 (35.0)	
Summer	45 (9.8)	27 (11.6)	13 (7.8)	3 (7.3)	2 (10.0)	
Wave 2 valid accelerometer data	*n* = 600	*n* = 307	*n* = 204	*n* = 58	*n* = 31	
Yes	420 (70.0)	213 (69.4)	151 (74.0)	35 (60.3)	21 (67.7)	0.233
Wave 2 season^a^	*n* = 419	*n* = 213	*n* = 151	*n* = 35	*n* = 21	
Autumn	99 (46.5)	69 (45.7)	14 (40.0)	8 (38.1)	99 (46.5)	0.549
Winter	50 (23.5)	30 (19.9)	8 (22.9)	6 (28.6)	50 (23.5)	
Spring	37 (17.4)	20 (13.3)	7 (20.0)	2 (9.5)	37 (17.4)	
Summer	27 (12.7)	32 (21.2)	6 (17.1)	5 (23.8)	27 (12.7)	
Wave 2 data collection occurred during COVID-19^b^	*n* = 420	*n* = 213	*n* = 155	*n* = 35	*n* = 21	
Yes	188 (44.8)	103 (48.4)	56 (37.1)	18 (51.4)	11 (52.4)	0.120
	Mean (SD)	Mean (SD)	Mean (SD)	Mean (SD)	Mean (SD)	*p*-value
Wave 1 accelerometer wear time (mins/day)	666.0 (64.5)	669.1 (67.3)	664.8 (63.5)	666.4 (54.5)	639.6 (56.6)	0.268
Wave 2 accelerometer wear time (mins/day)	884.9 (153.8)	893.0 (150.5)	886.1 (167.0)	852.4 (123.2)	848.8 (129.0)	0.347

^a^Based on end date of accelerometer wear. Denominator is the number of children with valid accelerometer data

^b^Based on end date of accelerometer wear being on or after the date Western Australia entered a State of Emergency (15/03/2020)

### Differences between dog ownership groups at wave 1

There were no differences at wave 1 for device-measured movement behaviours between dog ownership groups (all group main effects *p* > 0.05, see Additional File 4 for adjusted model coefficients). There were also no differences between groups for weekly structured physical activity or boys’ daily sleep. Weekly frequency of unstructured physical activity at wave 1 varied by dog ownership group. Girls in the dog owner group did 8.3 (95%CI 5.9, 10.7) more occasions of unstructured physical activity per week than non-dog owners and boys in the dog owner group did 6.8 (95%CI 4.4, 9.1) more occasions per week than non-dog owners. As well, girls in the dog loss group did 10.0 (95%CI 4.8, 15.1) more occasions per week of unstructured physical activity than non-dog owners and boys in the dog loss group did 11.3 (95%CI 6.7, 15.8) more occasions per week than non-dog owners. After excluding the dog walking and play items from unstructured physical activity, boys in the dog loss group did 5.9 (95%CI 1.9, 10.0) more occasions per week of unstructured physical activity than non-dog owners, but there were no other wave 1 between-group differences for this measure. In other words, the wave 1 between-group differences in total unstructured physical activity were mainly due to the addition of dog-facilitated physical activity among children who had a dog.

Screen time also differed at wave 1 between dog ownership groups: boys in the dog owner group had 27.5 (95%CI 8.6, 46.4) more minutes of daily screen time at wave 1 than boys in the non-dog owner group, while girls in the dog owner group had 26.0 (95%CI -44.9, -7.0) fewer minutes of daily screen time than girls in the non-dog owner group. Finally, girls in the dog acquired group had 0.5 (95%CI 0.0, 0.9) hours more daily sleep at wave 1 than non-dog owners.

### Change in movement behaviours

**Fig. 2:**
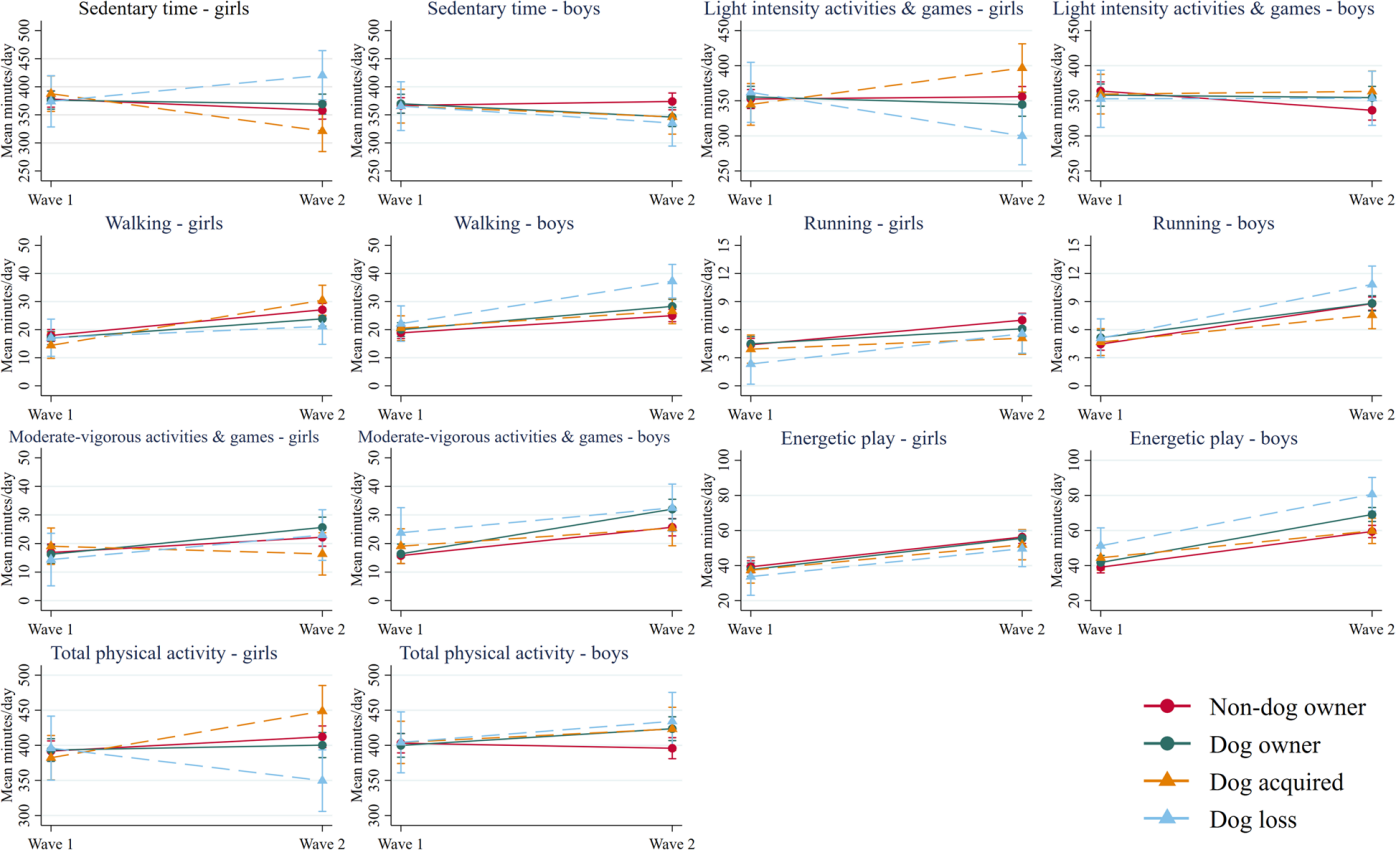
Estimated marginal means for device-measured movement behaviours at wave 1 and wave 2 by dog ownership group and child sex

**Fig. 3:**
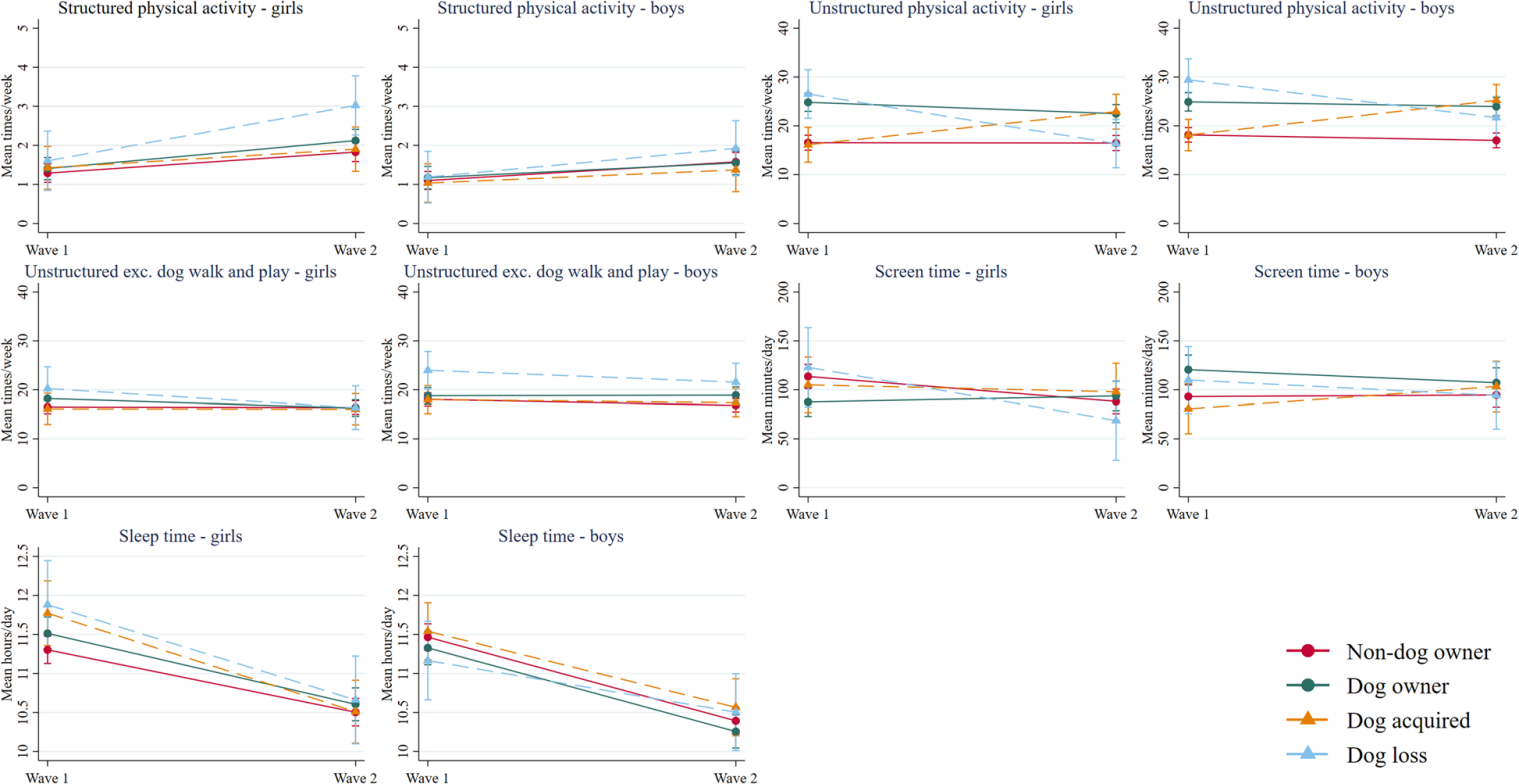
Estimated marginal means for parent-report movement behaviours at wave 1 and wave 2 by dog ownership group and child sex

Changes in movement behaviours by dog ownership group from preschool to fulltime school are displayed in Fig. 2 (device-measured) and Fig. 3 (parent-reported); estimated marginal means are reported in Tables 2, 3, 4 and 5. For girls, changes in movement behaviours varied by dog ownership group for sedentary time (group-by-time interaction *p* = 0.019), light intensity activities and games (*p* = 0.008), total physical activity (*p* = 0.019), unstructured physical activity (*p* < 0.001), and screen time (*p* = 0.007). For boys, significant group-by-time interactions were observed for energetic play (*p* = 0.049) and unstructured physical activity (*p* < 0.001). The group-by-time-by-sex interactions were significant for sedentary time, light intensity activities and games, walking, total physical activity, and screen time (all *p* < 0.05). Differences in change between dog ownership groups are described below; adjusted LMM coefficients are provided in Additional File 4.

**Table 2:** Estimated marginal means for girls’ device-measured movement behaviours by dog ownership group

Girls	Wave 1	Wave 2	Within-group change	Between-group difference in change
	Mean (95%CI)	Mean (95%CI)		
Sedentary time (mins/day)				*p* = 0.019
Non-dog owner	377.9 (363.5, 392.4)	357.6 (342.1, 373.0)	-20.4 (-42.8, 2.1)	Ref
Dog owner	376.4 (360.3, 392.5)	369.5 (351.6, 387.3)	-6.9 (-31.7, 17.8)	13.4 (-13.8, 40.6)
Dog acquired	387.4 (355.8, 419.0)	321.0 (284.4, 357.7)	-66.3 (-113.4, -19.2)	-46.0 (-94.7, 2.8)
Dog loss	373.7 (328.1, 419.3)	420.0 (376.1, 463.9)	46.3 (-14.8, 107.5)	66.7 (4.8, 128.6)
Light intensity activities and games (mins/day)				*p* = 0.008
Non-dog owner	352.5 (339.0, 366.1)	355.9 (341.4, 370.4)	3.3 (-17.7, 24.3)	Ref
Dog owner	355.6 (340.5, 370.7)	344.7 (328.0, 361.5)	-10.9 (-34.0, 12.2)	-14.3 (-39.7, 11.2)
Dog acquired	344.9 (315.2, 374.5)	396.8 (362.5, 431.2)	52.0 (7.9, 96.0)	48.6 (3.1, 94.2)
Dog loss	362.1 (319.3, 404.8)	300.0 (258.8, 341.2)	-62.1 (-119.3, -4.9)	-65.5 (-123.3, -7.6)
Walking (mins/day)				*p* = 0.051
Non-dog owner	17.9 (15.8, 20.0)	27.1 (24.9, 29.3)	9.2 (6.0, 12.4)	Ref
Dog owner	17.0 (14.6, 19.3)	23.9 (21.3, 26.5)	6.9 (3.3, 10.4)	-2.3 (-6.2, 1.6)
Dog acquired	14.4 (9.8, 19.0)	30.5 (25.2, 35.8)	16.1 (9.4, 22.8)	6.9 (0.0, 13.9)
Dog loss	17.1 (10.5, 23.8)	21.2 (14.8, 27.6)	4.0 (-4.7, 12.8)	-5.1 (-14.0, 3.7)
Running (mins/day)				*p* = 0.221
Non-dog owner	4.4 (3.7, 5.1)	7.0 (6.3, 7.7)	2.6 (1.6, 3.6)	Ref
Dog owner	4.5 (3.7, 5.3)	6.1 (5.2, 6.9)	1.6 (0.5, 2.7)	-1.0 (-2.2, 0.2)
Dog acquired	3.9 (2.4, 5.4)	5.1 (3.4, 6.8)	1.2 (-0.9, 3.3)	-1.5 (-3.6, 0.7)
Dog loss	2.3 (0.2, 4.5)	5.6 (3.5, 7.7)	3.2 (0.5, 6.0)	0.6 (-2.1, 3.4)
Moderate-vigorous activities and games (mins/day)				*p* = 0.128
Non-dog owner	16.9 (14.0, 19.8)	22.2 (19.1, 25.3)	5.3 (0.6, 10.0)	Ref
Dog owner	16.2 (13.0, 19.5)	25.6 (22.0, 29.2)	9.4 (4.2, 14.6)	4.1 (-1.7, 9.9)
Dog acquired	19.1 (12.7, 25.4)	16.4 (9.0, 23.8)	-2.7 (-12.6, 7.2)	-8.0 (-18.3, 2.3)
Dog loss	14.4 (5.2, 23.6)	23.0 (14.1, 31.8)	8.6 (-4.3, 21.5)	3.3 (-9.8, 16.3)
Energetic play (mins/day)				*p* = 0.948
Non-dog owner	39.3 (35.9, 42.7)	56.3 (52.6, 59.9)	17.0 (11.7, 22.2)	Ref
Dog owner	37.7 (33.9, 41.5)	55.5 (51.4, 59.7)	17.9 (12.1, 23.6)	0.9 (-5.5, 7.3)
Dog acquired	37.4 (30.0, 44.8)	51.9 (43.3, 60.5)	14.5 (3.4, 25.5)	-2.5 (-13.9, 8.9)
Dog loss	33.7 (23.1, 44.4)	49.7 (39.4, 60.0)	16.0 (1.6, 30.3)	-1.0 (-15.5, 13.5)
Total physical activity (mins/day)				*p* = 0.019
Non-dog owner	391.8 (377.4, 406.3)	412.2 (396.7, 427.6)	20.4 (-2.1, 42.8)	Ref
Dog owner	393.3 (377.3, 409.4)	400.3 (382.4, 418.1)	6.9 (-17.8, 31.7)	-13.4 (-40.6, 13.8)
Dog acquired	382.4 (350.7, 414.0)	448.7 (412.1, 485.3)	66.3 (19.2, 113.4)	46.0 (-2.8, 94.7)
Dog loss	396.0 (350.4, 441.6)	349.7 (305.8, 393.6)	-46.3 (-107.5, 14.8)	-66.7 (-128.6, -4.8)

Marginal means estimated from fully adjusted LMM (*n* = 537) which included group*time*sex interaction and lower order terms, child age, mother’s education, mother’s work status, having a yard big enough for running, dwelling type, length of follow-up, accelerometer data collection season, accelerometer data collected during COVID-19, and accelerometer wear time. Group*time interaction derived separately for boys and girls from the fully interacted model. Energetic play is the sum of walking, running, and moderate-to-vigorous activities and games. Total physical activity is the sum of light activities and games and energetic play

**Table 3:** Estimated marginal means for boys’ device-measured movement behaviours by dog ownership group

Boys	Wave 1	Wave 2	Within-group change	Between-group difference in change
	Mean (95%CI)	Mean (95%CI)		
Sedentary time (mins/day)				*p* = 0.117
Non-dog owner	366.7 (352.8, 380.5)	373.8 (358.9, 388.8)	7.1 (-14.3, 28.6)	Ref
Dog owner	369.8 (352.9, 386.7)	346.1 (329.1, 363.1)	-23.7 (-47.7, 0.2)	-30.9 (-58.4, -3.4)
Dog acquired	365.5 (335.4, 395.7)	346.4 (315.6, 377.3)	-19.1 (-61.4, 23.1)	-26.3 (-70.2, 17.7)
Dog loss	365.5 (322.1, 408.9)	335.5 (294.4, 376.6)	-30.0 (-87.1, 27.1)	-37.2 (-95.7, 21.4)
Light intensity activities and games (mins/day)				*p* = 0.184
Non-dog owner	363.9 (350.9, 376.9)	336.5 (322.4, 350.5)	-27.4 (-47.5, -7.4)	Ref
Dog owner	358.1 (342.3, 374.0)	354.6 (338.6, 370.5)	-3.5 (-25.9, 18.9)	23.9 (-1.8, 49.6)
Dog acquired	359.5 (331.2, 387.8)	363.5 (334.5, 392.4)	4.0 (-35.6, 43.5)	31.4 (-9.7, 72.4)
Dog loss	352.9 (312.1, 393.6)	353.6 (315.1, 392.2)	0.8 (-52.6, 54.1)	28.2 (-26.6, 82.9)
Walking (mins/day)				p = 0.165
Non-dog owner	18.9 (16.9, 20.9)	25.0 (22.8, 27.2)	6.1 (3.1, 9.2)	Ref
Dog owner	20.0 (17.6, 22.5)	28.3 (25.8, 30.8)	8.3 (4.8, 11.7)	2.1 (-1.8, 6.0)
Dog acquired	20.6 (16.2, 24.9)	26.6 (22.1, 31.1)	6.1 (0.0, 12.1)	-0.1 (-6.3, 6.2)
Dog loss	22.2 (15.8, 28.5)	37.3 (31.3, 43.3)	15.1 (7.0, 23.2)	9.0 (0.6, 17.3)
Running (mins/day)				*p* = 0.206
Non-dog owner	4.5 (3.8, 5.1)	8.8 (8.1, 9.5)	4.3 (3.4, 5.3)	Ref
Dog owner	5.1 (4.3, 6.0)	8.8 (8.0, 9.6)	3.7 (2.6, 4.7)	-0.7 (-1.9, 0.6)
Dog acquired	4.7 (3.2, 6.1)	7.6 (6.1, 9.0)	2.9 (1.0, 4.8)	-1.4 (-3.4, 0.5)
Dog loss	5.1 (3.0, 7.2)	10.8 (8.9, 12.8)	5.7 (3.2, 8.3)	1.4 (-1.2, 4.0)
Moderate-vigorous activities and games (mins/day)				*p* = 0.136
Non-dog owner	15.8 (13.0, 18.6)	25.7 (22.7, 28.7)	9.9 (5.4, 14.4)	Ref
Dog owner	16.4 (13.0, 19.8)	32.0 (28.6, 35.5)	15.7 (10.6, 20.7)	5.7 (-0.1, 11.6)
Dog acquired	19.1 (13.0, 25.2)	25.4 (19.2, 31.7)	6.3 (-2.5, 15.2)	-3.6 (-12.8, 5.7)
Dog loss	23.8 (15.0, 32.5)	32.5 (24.2, 40.8)	8.7 (-3.4, 20.8)	-1.2 (-13.7, 11.2)
Energetic play (mins/day)				*p* = 0.049
Non-dog owner	39.1 (35.8, 42.3)	59.4 (55.9, 62.9)	20.3 (15.3, 25.4)	Ref
Dog owner	41.8 (37.8, 45.7)	69.1 (65.1, 73.1)	27.3 (21.7, 32.9)	7.0 (0.5, 13.4)
Dog acquired	44.5 (37.4, 51.5)	59.8 (52.6, 67.0)	15.3 (5.4, 25.2)	-5.0 (-15.3, 5.3)
Dog loss	51.3 (41.1, 61.5)	80.6 (71.0, 90.3)	29.3 (16.0, 42.7)	9.0 (-4.7, 22.7)
Total physical activity (mins/day)				*p* = 0.117
Non-dog owner	403.0 (389.2, 416.9)	395.9 (380.9, 410.9)	-7.1 (-28.6, 14.3)	Ref
Dog owner	399.9 (383.0, 416.8)	423.7 (406.7, 440.7)	23.7 (-0.2, 47.7)	30.9 (3.4, 58.4)
Dog acquired	404.2 (374.1, 434.3)	423.3 (392.5, 454.2)	19.1 (-23.1, 61.4)	26.3 (-17.7, 70.2)
Dog loss	404.2 (360.8, 447.6)	434.2 (393.1, 475.4)	30.0 (-27.1, 87.1)	37.2 (-21.4, 95.7)

Marginal means estimated from fully adjusted LMM (*n* = 537) which included group*time*sex interaction and lower order terms, child age, mother’s education, mother’s work status, having a yard big enough for running, dwelling type, length of follow-up, accelerometer data collection season, accelerometer data collected during COVID-19, and accelerometer wear time. Group*time interaction derived separately for boys and girls from the fully interacted model. Energetic play is the sum of walking, running, and moderate-to-vigorous activities and games. Total physical activity is the sum of light activities and games and energetic play

**Table 4:** Estimated marginal means for girls’ parent-report movement behaviours by dog ownership group

Girls	Wave 1	Wave 2	Within-group difference	Between-group difference in change
	Mean (95%CI)	Mean (95%CI)	Mean (95%CI)	
Structured physical activity (times/week)				*p* = 0.332
Non-dog owner	1.3 (1.1, 1.5)	1.8 (1.6, 2.1)	0.5 (0.2, 0.9)	Ref
Dog owner	1.4 (1.1, 1.7)	2.1 (1.8, 2.4)	0.7 (0.3, 1.1)	0.2 (-0.3, 0.6)
Dog acquired	1.4 (0.9, 2.0)	1.9 (1.3, 2.5)	0.5 (-0.2, 1.2)	-0.1 (-0.8, 0.7)
Dog loss	1.6 (0.8, 2.4)	3.0 (2.3, 3.8)	1.4 (0.5, 2.4)	0.9 (-0.1, 1.9)
Unstructured physical activity (times/week)				*p* = < 0.001
Non-dog owner	16.5 (15.0, 18.1)	16.5 (14.9, 18.0)	-0.1 (-1.7, 1.6)	Ref
Dog owner	24.8 (23.0, 26.7)	22.5 (20.6, 24.4)	-2.3 (-4.2, -0.4)	-2.3 (-4.6, 0.0)
Dog acquired	16.1 (12.5, 19.7)	22.9 (19.3, 26.5)	6.8 (3.2, 10.3)	6.8 (3.1, 10.6)
Dog loss	26.5 (21.6, 31.5)	16.4 (11.4, 21.3)	-10.2 (-15.0, -5.3)	-10.1 (-15.1, -5.1)
Unstructured physical activity – excluding dog walking and play (times/week)				*p* = 0.181
Non-dog owner	16.5 (15.1, 17.9)	16.4 (15.0, 17.8)	-0.1 (-1.6, 1.4)	Ref
Dog owner	18.2 (16.6, 19.9)	16.3 (14.6, 17.9)	-1.9 (-3.7, -0.2)	-1.8 (-3.9, 0.3)
Dog acquired	16.1 (12.9, 19.3)	16.0 (12.9, 19.2)	-0.1 (-3.3, 3.2)	0.0 (-3.4, 3.5)
Dog loss	20.2 (15.8, 24.7)	16.3 (11.9, 20.8)	-3.9 (-8.4, 0.6)	-3.8 (-8.4, 0.8)
Screen time (mins/day)				*p* = 0.007
Non-dog owner	113.6 (101.3, 125.9)	88.2 (75.6, 100.7)	-25.4 (-40.0, -10.9)	Ref
Dog owner	87.6 (72.8, 102.4)	93.7 (78.7, 108.8)	6.1 (-11.0, 23.3)	31.5 (10.8, 52.3)
Dog acquired	105.1 (76.8, 133.5)	98.0 (68.7, 127.2)	-7.2 (-39.7, 25.4)	18.2 (-16.2, 52.7)
Dog loss	123.0 (82.4, 163.7)	68.6 (28.0, 109.3)	-54.4 (-100.1, -8.7)	-29.0 (-76.2, 18.2)
Sleep time (hours/day)				*p* = 0.275
Non-dog owner	11.3 (11.1, 11.5)	10.5 (10.3, 10.7)	-0.8 (-1.0, -0.6)	Ref
Dog owner	11.5 (11.3, 11.7)	10.6 (10.4, 10.8)	-0.9 (-1.2, -0.6)	-0.1 (-0.4, 0.2)
Dog acquired	11.8 (11.4, 12.2)	10.5 (10.1, 10.9)	-1.3 (-1.8, -0.8)	-0.5 (-1.0, 0.1)
Dog loss	11.9 (11.3, 12.4)	10.7 (10.1, 11.2)	-1.2 (-1.9, -0.5)	-0.4 (-1.1, 0.3)

Marginal means estimated from fully adjusted LMM (structured, unstructured, sleep time all n = 570; screen time n = 568) which included group*time*sex interaction and lower order terms, child age, mother’s education, mother’s work status, having a yard big enough for running, dwelling type, length of follow-up, survey data collection season, and survey data collected during COVID-19. Group*time interaction derived separately for boys and girls from the fully interacted model

**Table 5:** Estimated marginal means for boys’ parent-report movement behaviours by dog ownership group

Boys	Wave 1	Wave 2	Within-group difference	Between-group difference in change
	Mean (95%CI)	Mean (95%CI)	Mean (95%CI)	
Structured physical activity (times/week)				*p* = 0.872
Non-dog owner	1.1 (0.9, 1.3)	1.6 (1.3, 1.8)	0.5 (0.2, 0.8)	Ref
Dog owner	1.2 (0.9, 1.5)	1.6 (1.2, 1.9)	0.4 (0.0, 0.8)	-0.1 (-0.6, 0.4)
Dog acquired	1.0 (0.5, 1.5)	1.4 (0.8, 1.9)	0.3 (-0.3, 1.0)	-0.1 (-0.9, 0.6)
Dog loss	1.2 (0.5, 1.8)	1.9 (1.2, 2.6)	0.7 (-0.1, 1.6)	0.3 (-0.7, 1.2)
Unstructured physical activity (times/week)				*p* = < 0.001
Non-dog owner	18.2 (16.7, 19.6)	17.0 (15.5, 18.5)	-1.1 (-2.7, 0.5)	Ref
Dog owner	24.9 (23.0, 26.8)	24.0 (22.1, 25.8)	-1.0 (-2.9, 0.9)	0.2 (-2.2, 2.5)
Dog acquired	18.1 (14.9, 21.3)	25.2 (22.0, 28.5)	7.1 (3.9, 10.3)	8.3 (4.8, 11.7)
Dog loss	29.4 (25.1, 33.7)	21.7 (17.4, 26.0)	-7.7 (-12.0, -3.5)	-6.6 (-11.1, -2.1)
Unstructured physical activity – excluding dog walking and play (times/week)				*p* = 0.535
Non-dog owner	18.0 (16.7, 19.4)	16.8 (15.5, 18.2)	-1.2 (-2.7, 0.3)	Ref
Dog owner	18.8 (17.1, 20.5)	18.9 (17.2, 20.6)	0.1 (-1.6, 1.9)	1.3 (-0.8, 3.5)
Dog acquired	18.0 (15.1, 20.9)	17.4 (14.5, 20.2)	-0.6 (-3.6, 2.3)	0.6 (-2.6, 3.7)
Dog loss	24.0 (20.1, 27.8)	21.6 (17.7, 25.4)	-2.4 (-6.3, 1.5)	-1.2 (-5.3, 2.9)
Screen time (mins/day)				*p* = 0.135
Non-dog owner	93.1 (81.1, 105.0)	94.7 (82.4, 106.9)	1.6 (-12.7, 15.9)	Ref
Dog owner	120.5 (105.6, 135.5)	107.2 (92.0, 122.4)	-13.4 (-30.4, 3.7)	-15.0 (-36.1, 6.2)
Dog acquired	80.7 (55.2, 106.2)	103.4 (77.5, 129.2)	22.7 (-6.0, 51.4)	21.1 (-10.0, 52.2)
Dog loss	110.0 (75.7, 144.2)	94.2 (59.9, 128.6)	-15.7 (-53.7, 22.2)	-17.3 (-57.1, 22.4)
Sleep time (hours/day)				*p* = 0.620
Non-dog owner	11.5 (11.3, 11.6)	10.4 (10.2, 10.6)	-1.1 (-1.3, -0.9)	Ref
Dog owner	11.3 (11.1, 11.5)	10.3 (10.0, 10.5)	-1.1 (-1.3, -0.8)	0.0 (-0.3, 0.3)
Dog acquired	11.5 (11.2, 11.9)	10.6 (10.2, 10.9)	-1.0 (-1.4, -0.5)	0.1 (-0.4, 0.6)
Dog loss	11.2 (10.7, 11.7)	10.5 (10.0, 11.0)	-0.7 (-1.3, -0.1)	0.4 (-0.2, 1.0)

Marginal means estimated from fully adjusted LMM (structured, unstructured, sleep time all *n* = 570; screen time *n* = 568) which included group*time*sex interaction and lower order terms, child age, mother’s education, mother’s work status, having a yard big enough for running, dwelling type, length of follow-up, survey data collection season, and survey data collected during COVID-19. Group*time interaction derived separately for boys and girls from the fully interacted model

For girls’ light intensity activities and games, there were no differences in trajectories between the dog owner and non-dog owner groups. Acquiring a dog had a significant positive effect on girls’ change in light intensity activities and games (β = 48.6, 95%CI 3.1, 94.2) and losing a dog had a significant negative effect on girls’ change in light intensity activities and games (β = -65.5, 95%CI -123.3, -7.6). Overall, girls who acquired a dog increased their light intensity activities and games by 52.0 min/day (95%CI 7.9, 96.0) and girls who lost a dog decreased their light intensity activities and games by 62.1 min/day (95%CI -119.3, -4.9). There were no changes in light intensity activities and games for girls in the non-dog owner or dog owner groups.

For girls’ total physical activity, changes observed in the dog acquired and dog owner groups were not significantly different to the null change in the non-dog owner group. In contrast, losing a dog had a significant negative effect on girls’ change in total physical (β = -66.7, 95%CI -128.6, -4.8) and overall, the girl dog loss group decreased their total physical activity by 46.3 min/day (95%CI -107.5, 14.8). The changes for sedentary time were the inverse of changes in total physical activity, i.e., the increase in daily sedentary time for the girl dog loss group was significantly different to the non-dog owner group.

For boys, daily energetic play increased from preschool to school and the changes were similar for non-dog owner, dog acquired, and dog loss groups. Boy dog owners had a significantly greater increase in daily energetic play (β = 7.0, 95%CI 0.5, 13.4) and in total this group increased their daily energetic play by 27.3 min/day (95%CI 21.7, 32.9) compared to 20.3 min/day (95%CI 15.3, 25.4) in the non-dog owner group.

For unstructured physical activity, acquiring a dog had a significant positive effect on girls’ change in unstructured physical activity (β = 6.8, 95%CI 3.1, 10.6), while losing a dog had a significant negative effect on girls’ change in unstructured physical activity (β = -10.1, 95%CI -15.1, -5.1). Overall, the dog acquired group increased their weekly frequency of unstructured physical activity by 6.8 occasions/week (95%CI 3.2, 10.3) and the dog loss group decreased their weekly frequency by 10.2 occasions/week (95%CI -15.0, -5.3). Changes in boys’ unstructured physical activity were similar to those observed among girls. For both boys and girls, there were no significant group-by-time interactions observed after removing the dog walking and dog play items from the weekly frequency of unstructured physical activity, indicating changes were due to the addition of dog-facilitated physical activity among the dog acquired group and the loss of dog-facilitated physical activity among the dog loss group.

For girls’ daily screen time, no differences were observed between changes in the non-dog owner, dog acquired, and dog loss groups. The girl dog owner group had a significantly different trajectory to the non-dog owner group (β = 31.5, 95%CI 10.8, 52.3): girl dog owners had no change in daily screen time (6.1 min/day, 95%CI -11.0, 23.3) while girl non-dog owners reduced daily screen time by 25.4 min/day (95%CI -40.0, -10.9). As can be seen in Fig. 3, since the dog owner group had less daily screen time at wave 1, screen time was similar between groups at wave 2.

## Discussion

This study examined the effects of dog ownership, dog acquisition, and dog loss on children’s movement behaviours over a three-year period from preschool to fulltime school. Changes in some movement behaviours varied by dog ownership group and child sex. Specifically, dog acquisition increased girls’ daily time spent in light intensity activities and games and both girls’ and boys’ weekly frequency of unstructured physical activity. In contrast, losing a dog decreased girls’ daily time spent in light intensity physical activities and games and total physical activity, and decreased both girls’ and boys’ weekly frequency of unstructured physical activity. Overall, the findings suggest family dog acquisition has a positive effect on young children’s physical activity while losing a dog has a negative effect on young children’s physical activity.

Previous studies in children have been cross-sectional in design and unable to determine whether dog ownership is a cause or consequence of increased physical activity. The current study provides the first evidence that acquiring a dog may precede increases in certain types of children’s physical activity. This is important as it suggests there may be a causal relationship between dog ownership and physical activity in young children. Furthermore, the findings indicated differences between groups’ unstructured physical activity were driven by dog-facilitated physical activity. At both waves, children with a dog were accumulating 6–7 occasions per week of unstructured physical activity through dog walking and playing with the dog. The increase in unstructured physical activity for the dog acquired group and the decrease for the dog loss group were also only observed when including the items relating to children’s dog-facilitated physical activity. Therefore, dog acquisition could be a meaningful way to promote healthy movement behaviours in children and reduce their short and long-term risk of chronic disease. However, despite large absolute changes in total physical activity for the dog acquired groups, these did not reach statistical significance compared to changes in the non-dog owner groups.

Our findings support the limited evidence in adults finding dog acquisition was positively associated with increased physical activity [31, 33–36]. However, most of these studies had small samples [33–36], three did not adjust for confounders [33, 34, 36], and two had no comparison group [34, 36]. One large longitudinal study which employed a natural experiment similar to the current study found leisure-time walking increased by 31 min per week at 12-month follow-up after dog acquisition [31]. The only study with more than one year of follow-up [34] found very large increases in weekly leisure-time walking and total physical activity (> 4 h each) at three years following puppy acquisition, however there was no control group. In this study, girls who acquired a dog increased their participation light intensity activities and games by 52 min per day and girls and boys who acquired a dog increased their unstructured physical activity by 7 occasions per week. Given the lack of longitudinal studies in children and the methodological limitations of the handful of longitudinal studies conducted in adults, further longitudinal research with children and adults is needed to confirm the effects of dog acquisition on physical activity.

In contrast to the positive effects of dog acquisition on physical activity, losing a dog had a significant negative effect on physical activity, particularly for girls. It is difficult to compare these findings as there are no published studies examining the effects of losing a dog on physical activity among dog owners of any age. It is plausible the negative effects of dog loss on children’s physical activity could be related to the grieving process. Children, especially young children, can have strong attachment to the family dog [52, 53] and pets are often considered a member of the family [19, 54]. Losing a dog may be a child’s first experience of death and it can elicit a profound grief response [55]. Qualitative studies among children and adults report losing a companion animal is associated with avoidant coping strategies [56–58]. Thus, there may be decreased participation in activities that previously may have been done with the dog. Furthermore, dogs provide motivation and a sense of obligation for walking [32]. If family dog walking is not replaced with other activities after the loss of a dog, then it is reasonable that children’s physical activity levels may decline.

Being physically active is associated with better sleep [59] and more time being active may mean there is less time for screens, though evidence on the latter is inconsistent [49–51]. It is plausible dog owners could have less screen time and more sleep than non-dog owners due to increased time spent physically active. At the preschool time point, male dog owners had significantly greater daily screen time than non-dog owners, while female dog owners had significantly less daily screen time than non-dog owners. Over time, girls in the non-dog owner and dog loss groups reduced their screen time so at the school time point, daily screen time was similar across all groups. In contrast, boys’ daily screen time did not significantly change from preschool to school. Screen time findings in this study conflict with other research that has found only null associations between dog ownership and screen time [20, 21, 26, 28]. There were no differences between dog ownership groups for boys’ and girls’ sleep time, consistent with limited previous cross-sectional results reporting no association between dog ownership and children’s sleep [21], though one study found dog ownership was associated with increased sleep for children and adolescents during the COVID-19 pandemic [26]. Since the findings of the current study are inconsistent with hypotheses and conflict with some previous research, future studies should examine the mechanisms through which dog ownership could influence children’s screen time and sleep, including whether dog-facilitated physical activity replaces sedentary screen time or replaces other types of sedentary behaviours.

In this study, dog acquisition and dog loss differentially affected girls’ and boys’ movement behaviours. Movement behaviours in children are known to vary by sex [49–51], and cross-sectional research has also reported the effects of dog ownership differ by child sex. For example, an Australian study with children aged 10 to 12 years reported girl dog owners had greater total physical activity than non-owners, while boy dog owners had greater minutes of walking and total physical activity [20]. In contrast, other research observed positive associations between dog ownership and physical activity only among girls [23], and others have reported a greater proportion of young girls than boys play with their pet and walk their dog [25]. Future research should explore the potential mechanisms behind sex differences in physical activity related to dog ownership, including differences in the ways in which young girls and boys interact with their dogs. Future studies should also include sex interaction terms or stratify analyses by sex to better understand the effects of dog ownership on movement behaviours.

Cross-sectional research typically reports positive associations between dog ownership and children’s physical activity [20–25], however, in this study continuing dog owners did not participate in more physical activity (except for parent-reported unstructured physical activity) than non-dog owners. As such, there may be an attenuation of the effect of dog acquisition and ownership on physical activity over time, which was observed in the declining frequency of girl dog owners’ unstructured physical activity. Since this study did not have data available on the length of dog ownership or the timing of dog acquisition, we were unable to examine this further. Other longitudinal studies with multiple follow-ups and known timing of dog acquisition have shown adults’ physical activity increases following dog acquisition and then reduces with ongoing follow-up [33–35]. Owner physical activity may also be related to the age and/or health of the dog [32, 60] and other dog factors like size, breed, and energy levels have been correlated with owner physical activity [60–63]. Therefore, it is important future research considers these ‘dog demographics’ and length of dog ownership to better understand the relationship between dog ownership and movement behaviours in children and other population groups.

While we have identified associations between dog acquisition and increased physical activity, we cannot be certain these effects are due to physical activity that is done with the dog. There is some suggestion this may be the case, since the significant increase in parent-reported unstructured physical activity following dog acquisition was not observed after the dog-related items were excluded from the analysis. However, this needs to be explored further. Future studies would benefit from matching device-based physical activity measures to time-use diaries to specifically examine the contribution dog-facilitated physical activity makes to physical activity. Longitudinal comparisons between dog owning children who do and don’t undertake dog-facilitated physical activity would also be beneficial to extend findings from cross-sectional research [21, 25, 27]. Physical activity benefits related to dog ownership may only be observed among children who accumulate greater amounts of dog-facilitated physical activity, as long as the dog-facilitated physical activity is not replacing other types of physical activity. Other analytical approaches, such as compositional analysis [7, 9], may help to better understand how movement behaviours across the whole 24-h day are influenced by dog ownership.

### Strengths and limitations

A strength of this study is the natural experiment design and large sample of children used to examine the impact of dog ownership and changing dog ownership on device-based and parent-reported movement behaviours measured over three years. Other strengths include the adjustment for child and family sociodemographic characteristics; since dog ownership is selected into, it is important to adjust for confounding as factors that influence ownership may also be related to movement behaviours. This study also used validated machine learning accelerometer data processing methods to provide a rigorous approach to examining children’s daily movement behaviours.

This was a secondary analysis of existing data and thus it was constrained by the data collected in the original study; only dog ownership status was collected. One of the ways in which dog ownership may influence physical activity is through the human-animal bond, which likely has a greater influence on dog-facilitated physical activity than simply owning a dog [32, 64] and is a key factor in explaining dog walking behaviours among adults [32]. In children too, greater attachment to the family dog has been positively associated with increased physical activity [65].

We also had only two waves of data, and so could not determine if changes in movement behaviours associated with dog acquisition and dog loss were sustained. In addition, while the overall sample size was large, there were a small number of children among the dog acquired and dog loss groups which reduced the power to detect statistically significant effects. In particular, the dog loss groups had very wide confidence intervals for all measures, and so, despite some large absolute differences, few reached statistical significance. Additionally, there was substantial attrition in PLAYCE between wave 1 and wave 2 that may limit the generalisability of the findings. Finally, close to half the wave 2 data was collected during the COVID-19 pandemic, however, prior research has suggested Western Australian COVID-19 lockdowns did not affect young children’s physical activity [66].

We recommend future cohort studies, particularly birth cohorts, incorporate more dog-related measures in their studies, including the amount and intensity of dog-facilitated physical activity. Such longitudinal research would enable further understanding of the effects of dog ownership, dog acquisition, and dog loss on children’s movement behaviours and other developmental and health outcomes. Further research following children and families – that includes parent and sibling movement behaviours – for multiple follow-ups will also advance the field.

## Conclusion

Dog acquisition had a significant positive effect and dog loss had a significant negative effect on the change in young children’s movement behaviours over the transition from preschool to fulltime school. However, these effects were different for boys and girls and were not observed across all movement behaviours. Results from this study indicate the benefits of dog ownership begin early in childhood. Further longitudinal research is needed to confirm these results, and future studies should examine the specific contribution dog-facilitated physical activity makes to total physical activity.

### Supplementary Information

**Additional file 1.**

**Additional file 2.**

**Additional file 3.**

**Additional file 4.**

## Data Availability

The datasets used during the current study are available through application to the PLAYCE study.

## Abbreviations

ANOVA: Analysis of Variance
COVID-19: Coronavirus disease of 2019
ECEC: Early childhood education and care
LMM: Linear mixed effects model
PLAYCE: Play Spaces and Environments for Children’s Physical Activity
STROBE: Strengthening the reporting of observational studies in epidemiology
