# Supplementary material for: Longitudinal effects of dog ownership, dog acquisition, and dog loss on children’s movement behaviours: findings from the PLAYCE cohort study
Source: Int J Behav Nutr Phys Act. 2024 Jan 30;21:7. doi: 10.1186/s12966-023-01544-9 (PMC10826268; doi:10.1186/s12966-023-01544-9)
Supplement: Supplementary file 2 — Additional file 2. [file 12966_2023_1544_MOESM2_ESM.docx]

# Additional File 2

## PLAYCE study child characteristics

The characteristics for all children (n=1638 with valid wave 1 age and sex data), children who only participated in wave 1 (n=999 with valid wave 1 age and sex data), and children who participated in wave 1 and 2 (n=628 with valid wave 1 and 2 age and sex data) are reported in Additional Table 2. Children who only participated at wave 1 were older than children who participated in wave 1 and 2 (median 3.4 years vs. 3.2 years; Wilcoxon rank-sum test p<0.001). As well, compared to children with wave 1 and 2 data, a lower proportion of children who only participated at wave 1 had tertiary educated mothers (50.7% vs. 66.5%), lived in a standalone house (81.6% vs. 88.5%), and provided valid accelerometer data (62.1% vs. 76.9%; all Pearson chi-squared p<0.001).

Additional Table 2. Wave 1 characteristics of children in the PLAYCE study.

|  | All children | Wave 1 only | Wave 1 and 2 |  |
| --- | --- | --- | --- | --- |
|  | n=1638 | n=999 | n=628 |  |
|  | Median (IQR) | Median (IQR) | Median (IQR) | p-value |
| wave 1 age (years) | 3.3 (1.2) | 3.4 (1.2) | 3.2 (1.1) | <0.001 |
|  | n (%) | n (%) | n (%) | p-value |
| Child sex | n=1638 | n=999 | n=628 | 0.561 |
| Female | 782 (47.7) | 472 (47.3) | 306 (48.7) |  |
| Dog ownership | n=1628 | n=993 | n=625 | 0.169 |
| Yes | 682 (41.9) | 430 (43.3) | 249 (39.8) |  |
| Mother’s education | n=1620 | n=985 | n=624 | <0.001 |
| Secondary school, trade, diploma | 699 (43.2) | 486 (49.3) | 209 (33.5) |  |
| Tertiary degree | 921 (56.9) | 499 (50.7) | 415 (66.5) |  |
| Mother’s work status | n=1622 | n=986 | n=625 | 0.130 |
| Not in paid employment | 307 (18.9) | 188 (19.1) | 116 (18.6) |  |
| Working full-time | 526 (32.4) | 336 (34.1) | 186 (29.8) |  |
| Working part-time | 789 (48.6) | 462 (46.9) | 323 (51.7) |  |
| Yard size | n=1540 | n=928 | n=601 | 0.363 |
| Big enough for running and playing | 1321 (85.8) | 789 (85.0) | 521 (86.7) |  |
| Dwelling type | n=1562 | n=942 | n=609 | <0.001 |
| House | 1317 (84.3) | 769 (81.6) | 539 (88.5) |  |
| Duplex/townhouse/flat/other | 245 (15.7) | 173 (18.4) | 70 (11.5) |  |
| Wave 1 valid accelerometer data | n=1638 | n=999 | n=628 | <0.001 |
| Yes | 1111 (67.8) | 620 (62.1) | 483 (76.9) |  |
| Wave 1 season^1^ | n=1111 | n=620 | n=483 | 0.619 |
| Summer | 123 (11.1) | 73 (11.8) | 48 (9.9) |  |
| Autumn | 301 (27.1) | 162 (26.1) | 139 (28.8) |  |
| Winter | 287 (25.8) | 157 (25.3) | 126 (26.1) |  |
| Spring | 400 (36.0) | 228 (36.8) | 170 (35.2) |  |
|  | Mean (SD) | Mean (SD) | Mean (SD) | p-value |
| Wave 1 accelerometer wear time (mins/day) | 665.6 (64.6) | 664.8 (64.5) | 667.2 (64.9) | 0.540 |

Notes: Sample of children is based on having valid age and sex data. The sum of wave 1 only (n=999) and wave 1 and 2 children (n=628) does not equal the wave 1 total (n=1638) as 11 children who participated at wave 2 did not provide minimum data (age and sex).

^1^ Based on end date of accelerometer wear. Denominator is the number of children with valid accelerometer data.
