# Supplementary material for: Longitudinal effects of dog ownership, dog acquisition, and dog loss on children’s movement behaviours: findings from the PLAYCE cohort study
Source: Int J Behav Nutr Phys Act. 2024 Jan 30;21:7. doi: 10.1186/s12966-023-01544-9 (PMC10826268; doi:10.1186/s12966-023-01544-9)
Supplement: Supplementary file 3 — Additional file 3. [file 12966_2023_1544_MOESM3_ESM.docx]

# Additional File 3

## Unadjusted model coefficients

Unadjusted models included only the group*time*sex interaction and lower order terms. Unadjusted model coefficients are reported in Additional Table 3 for device-measured movement behaviours and Additional Table 4 for parent-reported movement behaviours. For ease of interpretation, coefficients were derived separately for girls and boys. A significant group*time*sex interaction was observed for sedentary time, walking, and screen time (all p<0.05). The group*time interaction was significant for girls’ sedentary time, girls’ and boys’ unstructured physical activity, and girls’ screen time (all p<0.05).

Additional Table 3. Unadjusted LMM estimated coefficients (β) of device-measured movement behaviours.

|  | Sedentary time | Light intensity activities and games | Walking | Running | Moderate-vigorous activities and games | Energetic play | Total physical activity |
| --- | --- | --- | --- | --- | --- | --- | --- |
|  | β (95% CI) | β (95% CI) | β (95% CI) | β (95% CI) | β (95% CI) | β (95% CI) | β (95% CI) |
| Girls |  |  |  |  |  |  |  |
| Dog owner | -11.8 (-45.7, 22.0) | 4.7 (-14.5, 23.9) | -1.1 (-3.9, 1.8) | 0.3 (-0.7, 1.2) | -0.9 (-4.8, 3.1) | -1.8 (-6.5, 2.8) | 2.9 (-17.4, 23.2) |
| Dog acquired | 19.1 (-39.6, 77.9) | -0.7 (-34.0, 32.5) | -3.5 (-8.4, 1.4) | -0.7 (-2.4, 1.0) | 1.5 (-5.3, 8.4) | -2.7 (-10.7, 5.4) | -3.3 (-38.5, 31.9) |
| Dog loss | -29.1 (-111.1, 52.9) | 5.7 (-40.7, 52.0) | -0.8 (-7.6, 6.0) | -2.5 (-4.8, -0.2) | -3.5 (-13.0, 6.1) | -7.0 (-18.2, 4.2) | -1.2 (-50.3, 47.9) |
| Wave 2 | 158.0 (125.9, 190.1) | 49.1 (31.5, 66.7) | 9.5 (6.9, 12.0) | 3.5 (2.7, 4.3) | 4.0 (0.2, 7.8) | 16.9 (12.6, 21.2) | 66.1 (47.4, 84.8) |
| Wave 2*dog owner | 24.0 (-25.3, 73.4) | -14.4 (-41.4, 12.5) | -2.4 (-6.3, 1.5) | -1.2 (-2.5, 0.0) | 3.5 (-2.4, 9.4) | -0.1 (-6.6, 6.5) | -14.6 (-43.2, 14.1) |
| Wave 2*dog acquired | -80.7 (-168.7, 7.2) | 32.9 (-15.2, 81.1) | 6.3 (-0.6, 13.3) | -1.6 (-3.9, 0.6) | -8.2 (-18.6, 2.3) | -3.5 (-15.2, 8.3) | 29.3 (-21.9, 80.5) |
| Wave 2*dog loss | 113.3 (1.7, 225.0) | -62.5 (-123.8, -1.3) | -5.4 (-14.2, 3.5) | 0.5 (-2.4, 3.4) | 2.2 (-11.0, 15.5) | -2.3 (-17.2, 12.6) | -65.0 (-130.1, 0.0) |
| Group p-value | 0.665 | 0.963 | 0.553 | 0.097 | 0.808 | 0.581 | 0.985 |
| Time*group p-value | 0.027 | 0.056 | 0.065 | 0.156 | 0.181 | 0.935 | 0.088 |
| Boys |  |  |  |  |  |  |  |
| Dog owner | -0.2 (-35.7, 35.2) | -4.4 (-24.5, 15.6) | 0.9 (-2.0, 3.9) | 0.6 (-0.4, 1.6) | 0.7 (-3.5, 4.8) | 2.4 (-2.4, 7.3) | -2.0 (-23.3, 19.2) |
| Dog acquired | -16.9 (-73.0, 39.3) | -9.2 (-41.0, 22.5) | 1.6 (-3.1, 6.3) | 0.1 (-1.5, 1.7) | 3.2 (-3.3, 9.8) | 5.1 (-2.6, 12.8) | -4.0 (-37.7, 29.6) |
| Dog loss | -27.1 (-105.1, 50.9) | -12.5 (-56.6, 31.7) | 3.0 (-3.5, 9.5) | 0.6 (-1.6, 2.8) | 7.9 (-1.2, 17.0) | 11.8 (1.1, 22.5) | -0.7 (-47.4, 46.0) |
| Wave 2 | 189.0 (157.7, 220.3) | 15.6 (-1.5, 32.7) | 6.4 (4.0, 8.9) | 5.0 (4.3, 5.8) | 8.7 (5.0, 12.4) | 20.2 (16.0, 24.4) | 35.7 (17.6, 53.9) |
| Wave 2*dog owner | -38.9 (-88.5, 10.8) | 19.2 (-7.9, 46.3) | 2.1 (-1.8, 6.0) | -0.8 (-2.1, 0.5) | 5.1 (-0.8, 11.0) | 6.1 (-0.5, 12.7) | 25.2 (-3.6, 54.0) |
| Wave 2*dog acquired | -52.1 (-131.4, 27.1) | 32.0 (-11.5, 75.5) | 0.5 (-5.8, 6.8) | -1.3 (-3.4, 0.7) | -3.0 (-12.4, 6.4) | -3.7 (-14.3, 6.9) | 28.3 (-17.9, 74.5) |
| Wave 2*dog loss | -104.3 (-210.4, 1.7) | 19.4 (-38.4, 77.3) | 9.1 (0.8, 17.5) | 1.5 (-1.2, 4.2) | -0.9 (-13.5, 11.7) | 9.6 (-4.5, 23.7) | 29.1 (-32.4, 90.6) |
| Group p-value | 0.853 | 0.862 | 0.739 | 0.674 | 0.317 | 0.116 | 0.995 |
| Time*group p-value | 0.121 | 0.345 | 0.164 | 0.198 | 0.247 | 0.120 | 0.273 |
| Time*group*sex p-value | 0.015 | 0.126 | 0.018 | 0.945 | 0.861 | 0.444 | 0.069 |

Notes: Unadjusted LMM (n=537) which included group*time*sex interaction and lower order terms. Coefficients reported separately for boys and girls. Energetic play is the sum of walking, running, and moderate-to-vigorous activities and games. Total physical activity is the sum of light activities and games and energetic play.

Additional Table 4. Unadjusted LMM estimated coefficients (β) of parent-reported movement behaviours.

|  | Structured physical activity (n=570) | Unstructured physical activity (n=570) | Unstructured physical activity exc. dog walk and play (n=570) | Screen time (n=568) | Sleep time (n=570) |
| --- | --- | --- | --- | --- | --- |
|  | β (95% CI) | β (95% CI) | β (95% CI) | β (95% CI) | β (95% CI) |
| Girls |  |  |  |  |  |
| Dog owner | 0.0 (-0.3, 0.4) | 8.5 (6.1, 10.9) | 2.0 (-0.2, 4.1) | -26.1 (-45.0, -7.2) | 0.2 (-0.1, 0.5) |
| Dog acquired | 0.0 (-0.6, 0.6) | 0.0 (-3.9, 3.9) | 0.0 (-3.5, 3.5) | -7.7 (-38.8, 23.3) | 0.6 (0.1, 1.0) |
| Dog loss | 0.2 (-0.6, 1.0) | 10.6 (5.4, 15.8) | 4.4 (-0.3, 9.1) | 15.2 (-27.5, 57.9) | 0.7 (0.1, 1.4) |
| Wave 2 | 0.6 (0.3, 0.8) | 0.0 (-1.4, 1.5) | 0.0 (-1.3, 1.3) | -22.7 (-36.0, -9.4) | -1.0 (-1.3, -0.8) |
| Wave 2*dog owner | 0.2 (-0.2, 0.7) | -2.1 (-4.4, 0.2) | -1.7 (-3.8, 0.4) | 30.3 (9.4, 51.1) | -0.1 (-0.4, 0.3) |
| Wave 2*dog acquired | 0.0 (-0.7, 0.8) | 7.0 (3.3, 10.8) | 0.2 (-3.2, 3.7) | 17.0 (-17.7, 51.6) | -0.4 (-1.0, 0.1) |
| Wave 2*dog loss | 0.9 (-0.1, 1.9) | -10.0 (-15.0, -5.0) | -3.7 (-8.3, 0.9) | -28.8 (-76.3, 18.7) | -0.4 (-1.1, 0.3) |
| Group p-value | 0.961 | <0.001 | 0.115 | 0.033 | 0.019 |
| Time*group p-value | 0.313 | <0.001 | 0.210 | 0.011 | 0.413 |
| Boys |  |  |  |  |  |
| Dog owner | 0.0 (-0.4, 0.3) | 7.1 (4.7, 9.5) | 1.1 (-1.0, 3.3) | 29.6 (10.7, 48.5) | -0.1 (-0.4, 0.2) |
| Dog acquired | -0.1 (-0.6, 0.5) | 0.2 (-3.3, 3.8) | 0.2 (-3.0, 3.4) | -6.0 (-34.3, 22.2) | 0.0 (-0.4, 0.4) |
| Dog loss | 0.0 (-0.7, 0.7) | 11.7 (7.1, 16.3) | 6.4 (2.2, 10.5) | 16.1 (-20.5, 52.7) | -0.2 (-0.8, 0.3) |
| Wave 2 | 0.5 (0.2, 0.8) | -0.9 (-2.4, 0.5) | -1.0 (-2.3, 0.3) | 3.3 (-9.8, 16.4) | -1.3 (-1.5, -1.1) |
| Wave 2*dog owner | 0.0 (-0.5, 0.4) | 0.0 (-2.3, 2.3) | 1.2 (-0.9, 3.3) | -14.2 (-35.3, 6.8) | 0.1 (-0.2, 0.4) |
| Wave 2*dog acquired | -0.1 (-0.9, 0.6) | 8.4 (5.0, 11.9) | 0.8 (-2.4, 3.9) | 18.7 (-12.5, 49.8) | 0.1 (-0.4, 0.7) |
| Wave 2*dog loss | 0.2 (-0.7, 1.1) | -6.8 (-11.3, -2.4) | -1.4 (-5.5, 2.7) | -14.5 (-54.4, 25.4) | 0.4 (-0.3, 1.1) |
| Group p-value | 0.992 | <0.001 | 0.024 | 0.011 | 0.708 |
| Time*group p-value | 0.937 | <0.001 | 0.549 | 0.203 | 0.639 |
| Time*group*sex p-value | 0.725 | 0.537 | 0.291 | 0.016 | 0.250 |

Notes: Unadjusted LMM which included group*time*sex interaction and lower order terms. Coefficients reported separately for boys and girls.
