# Supplementary material for: Longitudinal effects of dog ownership, dog acquisition, and dog loss on children’s movement behaviours: findings from the PLAYCE cohort study
Source: Int J Behav Nutr Phys Act. 2024 Jan 30;21:7. doi: 10.1186/s12966-023-01544-9 (PMC10826268; doi:10.1186/s12966-023-01544-9)
Supplement: Supplementary file 4 — Additional file 4. [file 12966_2023_1544_MOESM4_ESM.docx]

# Additional File 4

## Adjusted model coefficients

Adjusted model coefficients for girls and boys are reported in Additional Table 5 for device-measured movement behaviours and Additional Table 6 for parent-reported movement behaviours. For ease of interpretation, coefficients were derived separately for girls and boys from the model including the group-by-time-by-sex interaction term and all lower order interactions. The effects of dog ownership group on changes in movement behaviours varied by sex for sedentary time, light intensity activities and games, walking, total physical activity, and screen time (group*time*sex interaction p<0.05). The group*time interaction was significant for girls’ sedentary time, light intensity activities and games, total physical activity, unstructured physical activity, and screen time (all p<0.05). The group*time interaction was significant for boys’ energetic play and unstructured physical activity (both p<0.05).

Additional Table 5. Adjusted LMM estimated coefficients (β) of device-measured movement behaviours.

|  | Sedentary time | Light intensity activities and games | Walking | Running | Moderate-vigorous activities and games | Energetic play | Total physical activity |
| --- | --- | --- | --- | --- | --- | --- | --- |
|  | β (95% CI) | β (95% CI) | β (95% CI) | β (95% CI) | β (95% CI) | β (95% CI) | β (95% CI) |
| Girls |  |  |  |  |  |  |  |
| Dog owner | -1.5 (-21.1, 18.1) | 3.1 (-15.3, 21.5) | -0.9 (-3.8, 1.9) | 0.1 (-0.8, 1.0) | -0.6 (-4.6, 3.3) | -1.6 (-6.2, 3.0) | 1.5 (-18.1, 21.1) |
| Dog acquired | 9.5 (-24.1, 43.0) | -7.7 (-39.1, 23.8) | -3.5 (-8.4, 1.4) | -0.5 (-2.1, 1.1) | 2.2 (-4.6, 8.9) | -1.9 (-9.8, 6.0) | -9.5 (-43.0, 24.1) |
| Dog loss | -4.2 (-51.0, 42.6) | 9.5 (-34.3, 53.4) | -0.8 (-7.6, 6.0) | -2.0 (-4.3, 0.2) | -2.5 (-11.9, 6.9) | -5.6 (-16.5, 5.4) | 4.2 (-42.6, 51.0) |
| Wave 2 | -20.4 (-42.8, 2.1) | 3.3 (-17.7, 24.3) | 9.2 (6.0, 12.4) | 2.6 (1.6, 3.6) | 5.3 (0.6, 10.0) | 17.0 (11.7, 22.2) | 20.4 (-2.1, 42.8) |
| Wave 2*dog owner | 13.4 (-13.8, 40.6) | -14.3 (-39.7, 11.2) | -2.3 (-6.2, 1.6) | -1.0 (-2.2, 0.2) | 4.1 (-1.7, 9.9) | 0.9 (-5.5, 7.3) | -13.4 (-40.6, 13.8) |
| Wave 2*dog acquired | -46.0 (-94.7, 2.8) | 48.6 (3.1, 94.2) | 6.9 (0.0, 13.9) | -1.5 (-3.6, 0.7) | -8.0 (-18.3, 2.3) | -2.5 (-13.9, 8.9) | 46.0 (-2.8, 94.7) |
| Wave 2*dog loss | 66.7 (4.8, 128.6) | -65.5 (-123.3, -7.6) | -5.1 (-14.0, 3.7) | 0.6 (-2.1, 3.4) | 3.3 (-9.8, 16.3) | -1.0 (-15.5, 13.5) | -66.7 (-128.6, -4.8) |
| Group p-value | 0.937 | 0.894 | 0.554 | 0.283 | 0.823 | 0.723 | 0.937 |
| Time*group p-value | 0.019 | 0.008 | 0.051 | 0.221 | 0.128 | 0.948 | 0.019 |
| Boys |  |  |  |  |  |  |  |
| Dog owner | 3.1 (-17.5, 23.7) | -5.8 (-25.1, 13.5) | 1.2 (-1.8, 4.2) | 0.7 (-0.3, 1.7) | 0.6 (-3.5, 4.8) | 2.7 (-2.1, 7.5) | -3.1 (-23.7, 17.5) |
| Dog acquired | -1.2 (-33.3, 30.9) | -4.4 (-34.5, 25.7) | 1.7 (-3.0, 6.4) | 0.2 (-1.3, 1.7) | 3.3 (-3.2, 9.8) | 5.4 (-2.1, 12.9) | 1.2 (-30.9, 33.3) |
| Dog loss | -1.2 (-46.0, 43.6) | -11.0 (-53.1, 31.0) | 3.3 (-3.2, 9.8) | 0.6 (-1.5, 2.8) | 8.0 (-1.0, 17.0) | 12.2 (1.7, 22.7) | 1.2 (-43.6, 46.0) |
| Wave 2 | 7.1 (-14.3, 28.6) | -27.4 (-47.5, -7.4) | 6.1 (3.1, 9.2) | 4.3 (3.4, 5.3) | 9.9 (5.4, 14.4) | 20.3 (15.3, 25.4) | -7.1 (-28.6, 14.3) |
| Wave 2*dog owner | -30.9 (-58.4, -3.4) | 23.9 (-1.8, 49.6) | 2.1 (-1.8, 6.0) | -0.7 (-1.9, 0.6) | 5.7 (-0.1, 11.6) | 7.0 (0.5, 13.4) | 30.9 (3.4, 58.4) |
| Wave 2*dog acquired | -26.3 (-70.2, 17.7) | 31.4 (-9.7, 72.4) | -0.1 (-6.3, 6.2) | -1.4 (-3.4, 0.5) | -3.6 (-12.8, 5.7) | -5.0 (-15.3, 5.3) | 26.3 (-17.7, 70.2) |
| Wave 2*dog loss | -37.2 (-95.7, 21.4) | 28.2 (-26.6, 82.9) | 9.0 (0.6, 17.3) | 1.4 (-1.2, 4.0) | -1.2 (-13.7, 11.2) | 9.0 (-4.7, 22.7) | 37.2 (-21.4, 95.7) |
| Group p-value | 0.989 | 0.914 | 0.674 | 0.578 | 0.293 | 0.082 | 0.989 |
| Time*group p-value | 0.117 | 0.184 | 0.165 | 0.206 | 0.136 | 0.049 | 0.117 |
| Time*group*sex p-value | 0.013 | 0.023 | 0.014 | 0.957 | 0.843 | 0.425 | 0.013 |

Notes: Fully adjusted LMM (n=537) which included group*time*sex interaction and lower order terms, child age, mother’s education, mother’s work status, having a yard big enough for running, dwelling type, length of follow-up, accelerometer data collection season, accelerometer data collected during COVID-19, and accelerometer wear time. Coefficients reported separately for boys and girls. Energetic play is the sum of walking, running, and moderate-to-vigorous activities and games. Total physical activity is the sum of light activities and games and energetic play.

Additional Table 6. Adjusted LMM estimated coefficients (β) of parent-reported movement behaviours.

|  | Structured physical activity (n=570) | Unstructured physical activity (n=570) | Unstructured physical activity exc. dog walk and play (n=570) | Screen time (n=568) | Sleep time (n=570) |
| --- | --- | --- | --- | --- | --- |
|  | β (95% CI) | β (95% CI) | β (95% CI) | β (95% CI) | β (95% CI) |
| Girls |  |  |  |  |  |
| Dog owner | 0.1 (-0.2, 0.5) | 8.3 (5.9, 10.7) | 1.7 (-0.4, 3.8) | -26.0 (-44.9, -7.0) | 0.2 (-0.1, 0.5) |
| Dog acquired | 0.1 (-0.5, 0.7) | -0.4 (-4.3, 3.4) | -0.4 (-3.8, 3.1) | -8.5 (-39.0, 22.0) | 0.5 (0.0, 0.9) |
| Dog loss | 0.3 (-0.5, 1.1) | 10.0 (4.8, 15.1) | 3.8 (-0.9, 8.4) | 9.4 (-32.7, 51.6) | 0.6 (0.0, 1.2) |
| Wave 2 | 0.5 (0.2, 0.9) | -0.1 (-1.7, 1.6) | -0.1 (-1.6, 1.4) | -25.4 (-40.0, -10.9) | -0.8 (-1.0, -0.6) |
| Wave 2*dog owner | 0.2 (-0.3, 0.6) | -2.3 (-4.6, 0.0) | -1.8 (-3.9, 0.3) | 31.5 (10.8, 52.3) | -0.1 (-0.4, 0.2) |
| Wave 2*dog acquired | -0.1 (-0.8, 0.7) | 6.8 (3.1, 10.6) | 0.0 (-3.4, 3.5) | 18.2 (-16.2, 52.7) | -0.5 (-1.0, 0.1) |
| Wave 2*dog loss | 0.9 (-0.1, 1.9) | -10.1 (-15.1, -5.1) | -3.8 (-8.4, 0.8) | -29.0 (-76.2, 18.2) | -0.4 (-1.1, 0.3) |
| Group p-value | 0.819 | <0.001 | 0.184 | 0.045 | 0.048 |
| Time*group p-value | 0.332 | <0.001 | 0.181 | 0.007 | 0.275 |
| Boys |  |  |  |  |  |
| Dog owner | 0.1 (-0.3, 0.4) | 6.8 (4.4, 9.1) | 0.7 (-1.4, 2.9) | 27.5 (8.6, 46.4) | -0.1 (-0.4, 0.1) |
| Dog acquired | -0.1 (-0.6, 0.5) | 0.0 (-3.6, 3.5) | -0.1 (-3.2, 3.1) | -12.4 (-40.3, 15.5) | 0.1 (-0.3, 0.5) |
| Dog loss | 0.1 (-0.6, 0.8) | 11.3 (6.7, 15.8) | 5.9 (1.9, 10.0) | 16.9 (-19.3, 53.1) | -0.3 (-0.8, 0.2) |
| Wave 2 | 0.5 (0.2, 0.8) | -1.1 (-2.7, 0.5) | -1.2 (-2.7, 0.3) | 1.6 (-12.7, 15.9) | -1.1 (-1.3, -0.9) |
| Wave 2*dog owner | -0.1 (-0.6, 0.4) | 0.2 (-2.2, 2.5) | 1.3 (-0.8, 3.5) | -15.0 (-36.1, 6.2) | 0.0 (-0.3, 0.3) |
| Wave 2*dog acquired | -0.1 (-0.9, 0.6) | 8.3 (4.8, 11.7) | 0.6 (-2.6, 3.7) | 21.1 (-10.0, 52.2) | 0.1 (-0.4, 0.6) |
| Wave 2*dog loss | 0.3 (-0.7, 1.2) | -6.6 (-11.1, -2.1) | -1.2 (-5.3, 2.9) | -17.3 (-57.1, 22.4) | 0.4 (-0.2, 1.0) |
| Group p-value | 0.958 | <0.001 | 0.039 | 0.011 | 0.496 |
| Time*group p-value | 0.872 | <0.001 | 0.535 | 0.135 | 0.620 |
| Time*group*sex p-value | 0.739 | 0.440 | 0.214 | 0.011 | 0.190 |

Notes: Fully adjusted LMM which included group*time*sex interaction and lower order terms, child age, mother’s education, mother’s work status, having a yard big enough for running, dwelling type, length of follow-up, survey data collection season, and survey data collected during COVID-19. Coefficients reported separately for boys and girls.
